# Brain structures and functional connectivity associated with individual differences in trait proactive aggression

**DOI:** 10.1038/s41598-019-44115-4

**Published:** 2019-05-22

**Authors:** Wenfeng Zhu, Xiaolin Zhou, Ling-Xiang Xia

**Affiliations:** 1 Research Center of Psychology and Social Development, Southwest University, 400715 Chongqing, China; 2 School of Psychological and Cognitive Sciences, Peking University, 100871 Beijing, China; 3 Beijing Key Laboratory of Behavior and Mental Health, Peking University, 100871 Beijing, China; 4 PKU-IDG/McGovern Institute for Brain Research, Peking University, 100871 Beijing, China

**Keywords:** Social behaviour, Personality

## Abstract

Although considerable efforts have been made to understand the neural underpinnings of (state) reactive aggression, which is triggered by provocation or perceived threat, little is known about the neural correlates of proactive aggression, which is driven by instrumental motivations to obtain personal gains through aggressive means and which varies dramatically across individuals in terms of tendency of appealing to such means. Here, by combining structural (grey matter density, GMD) and functional (resting-state functional connection, RSFC) fMRI, we investigated brain structures and functional networks related to trait proactive aggression. We found that individual differences in trait proactive aggression were positively associated with GMD in bilateral dorsolateral prefrontal cortex (DLPFC) and negatively correlated with GMD in posterior cingulate cortex (PCC); they were also negatively correlated with the strength of functional connectivity between left PCC and other brain regions, including right DLPFC, right IPL, right MPFC/ACC, and bilateral precuneus. These findings shed light on the differential brain bases of proactive and reactive aggressions and suggested the neural underpinnings of proactive aggression.

## Introduction

Aggression refers to behavior that is carried out with an intention to cause physical or psychological harm to other individuals who are motivated to avoid the harm^1,2^. It has negative influence on individuals’ health and social relationships and can lead to considerable psychological or physical costs when aggressive behavior is expressed in exaggeration^3^. Aggression can be categorized into different categories along various dimensions. According to one common classification, reactive/impulsive aggression is triggered by provocation and/or perceived threat, whereas proactive/instrumental aggression is driven by instrumental motivations to achieve personal goals or to obtain personal gains through aggressive means with prior deliberation^1,4–6^. Two aspects of aggression, trait and state, can be further categorized correspondingly for the two types of aggression. While state reactive or proactive aggression is an aggressive response triggered by a specific provocation or incentive, trait reactive or proactive aggression refers to disposition that individuals tend to conduct reactive or proactive aggressive behavior in daily life across times and situations^7–9^.

Previous studies have shown that these two types of aggression differ in their psychological, physiological, and biological manifestations as well as in etiology^3,6,10,11^. For example, reactive aggression, but not proactive aggression, is associated with hostile attributional biases^12,13^. Individuals with high reactive aggression scores in relevant questionnaires tend to link ambiguous provocation with hostile intentions^14^. Proactive aggression, but not reactive aggression, is positively correlated with positive expectation of outcomes that aggressive behavior would result in^15,16^. Individuals with high proactive aggression scores are more likely to overvalue the outcome of aggression. Increasing neural activity of right dorsolateral prefrontal cortex (DLPFC) with anodal transcranial direct current stimulation (tDCS) can reduce the proactive aggression but not reactive aggression in males^4^.

Previous neural studies have been focused mostly on state reactive aggression, measuring participants’ responses to provocation in controlled experimental tasks, such as the Taylor Aggression Paradigm (TAP) and the Point Subtraction Aggression Paradigm (PSAP)^9,17–20^. The results suggest that brain regions involved in state reactive aggression include orbitofrontal cortex (OFC), ventromedial prefrontal cortex (VMPFC), anterior cingulate cortex (ACC), dorsolateral prefrontal cortex (DLPFC), superior temporal gyrus, and amygdala. Both behavioral^3,21,22^ and brain imaging studies demonstrate that emotion processing is crucially involved in state reactive aggression.

Comparatively, there are only a few studies investigating the neural basis of proactive aggression^18,23,24^. By increasing neural activity of right frontal cortex or inhibiting neural activity of left frontal cortex with brain stimulation technologies (tDCS and continuous theta-burst magnetic stimulation, cTBS), two studies induced right fronto-hemispheric dominance to explore the causal relationship between DLPFC and proactive aggressive behaviour measured by TAP and PSAP^4,23^. The proactive aggression was reduced after increasing neural activity of right frontal cortex in men^18^ and was increased after inhibiting neural activity of left frontal cortex, compared with the one after inhibiting neural activity of right frontal cortex^23^. But compared with the one after sham stimulation, the proactive aggression had not changed after inhibiting neural activity of left and right frontal cortex^23^. A third study, more similar to the current one, explored the brain structures responsible for trait proactive aggression in an adolescent sample^24^. The authors recruited 104 14-year-old adolescent twins and measured their brain structural MRI signals for tensor-based morphometry (TBM) and cortical thickness. After scanning, the authors asked participants to fill out Reactive-Proactive Aggression Questionnaire (RPQ)^5^, which contained items tapping into the prepotencies of trait proactive and reactive aggression, respectively. These items cover a broad spectrum of daily activities, including both verbal and physical threats and actions. Respondents were asked to evaluate how often such behaviours occurred to them. Across participants, the authors observed positive correlations between the total aggression scores and volumes of left caudate nuclei, bilateral putamen and right lateral orbitofrontal cortex, and between the total aggression scores and cortical thickness of superior temporal gyrus (STG), bilateral inferior temporal gyri (ITG), right middle temporal gyrus (MTG), right superior parietal lobe (SPL), bilateral inferior parietal lobes (IPL), and bilateral occipital lobes. They also observed a negative correlation between the total scores and right middle frontal cortex (MFC) in both TBM and cortical thickness. In post hoc analyses, authors additionally found that proactive aggression was positively correlated with volumes of left caudate, left putamen and right orbitofrontal cortex, and cortical thickness of right STG, right STG, left ITG and left paracentral gyrus, and was negatively correlated with volumes of right middle frontal cortex, cortical thickness of bilateral superior frontal cortex (SFC), bilateral MFC and left anterior cingulate cortex (ACC). These studies suggest that proactive aggression is correlated with grey matter structure and brain function of prefrontal cortex (DLPFC, OFC), parietal lobe (e.g. IPL and SPL), and cingulate cortex (e.g. ACC). And these regions have been found to be involved in the key aspects of trait proactive aggression as discussed below.

Individual differences in trait proactive aggression may comprise at least three aspects: (1) proactive aggressive motivation, which refers to approach motivation to attain instrumental goals through aggressive means^4,25^; (2) the ability and tendency of behavioral execution and monitoring (e.g., goal-orienting, planning, & premeditation)^26–28^; and (3) the abilities and tendencies of moral disinhibitions for proactive aggressive behavior, such as ability or tendency of moral disengagement, low moral cognitions and emotions^29^. Accordingly, we expected to find individual differences in brain structure or activity related to these three aspects of trait proactive aggression.

Firstly, individuals with stronger trait proactive aggression may have higher approach motivation. In RPQ, this approach motivation is measured by items like “used physical force to get others to do what you want”. Given that approach motivation involves left dorsolateral prefrontal cortex (DLPFC)^28,30^, it is likely that we would observe individual differences in DLPFC.

Secondly, compared with low proactive aggressive individuals, high proactive aggressive individuals exhibit more “cool-blooded”, organized, and planned aggressive behaviors in non-provoking contexts^25,26,28^. In RPQ, items like “carried a weapon to use in a fight” are related to this type of goal-driven behavior. Bilateral DLPFC plays a critical role in executive control^31–33^; harming others for self-gain activates regions including DLPFC, insula, and temporoparietal junction (TPJ) extending into the posterior STS^34^. Thus, we predicted that individual differences in trait proactive aggression could also involve DLPFC and some other regions.

Thirdly, highly proactive aggressive individuals typically have ability or tendency of low level of moral cognition and emotion, including lack of empathy^35–37^, theory of mind and guilt^12,35,38,39^. These individuals tend to use strategies such as moral disengagement to relieve or avoid moral inhibition (e.g., self-criticism) when approving proactive aggression^40–42^. Although items in RPQ did not describe the immoral features of high trait proactive aggression directly, given that moral disinhibition underlies the proactive aggressive behaviors, we predicted that brain regions involved in empathy, theory of mind and morality, such as ventral medial prefrontal cortex (VMPFC), precuneus, anterior cingulate cortex (ACC), posterior cingulate cortex (PCC), and temporoparietal junction (TPJ)^43,44^, could also exhibit individual differences regarding the moral aspect of trait proactive aggression.

In the current study, we continued this line of prior researches by focusing on adult participants and by examining the neuroanatomical feature and functional networks underlying the individual differences in trait proactive aggression. We collected structural imaging data from 240 participants and resting-state functional imaging data from 162 (out of the 240) participants and examined the correlations between the brain measures and the trait proactive (and reactive) aggression scores on RPQ.

## Results

### Descriptive data

Table 1 shows the mean scores and SDs for reactive and proactive aggression and the age of the males and females for the 240 participants. The differences between females and males in proactive aggression (*F* = 1.43, *p* = 0.23), reactive aggression (*F* = 0.15, *p* = 0.70) or age (*F* = 0.21, *p* = 0.65) were not statistically significant.

**Table 1:** Demographic and behavioral data (n = 240).

	Males		Females	
	Means	SD	Means	SD
Age	20.32	1.851	20.31	2.03
proactive aggression	1.07	2.40	0.92	1.84
reactive aggression	8.74	4.21	8.18	4.32

Note: n = number; SD = standard deviation.

### Correlation of regional GMD with scores of proactive aggression

**Figure 1:**
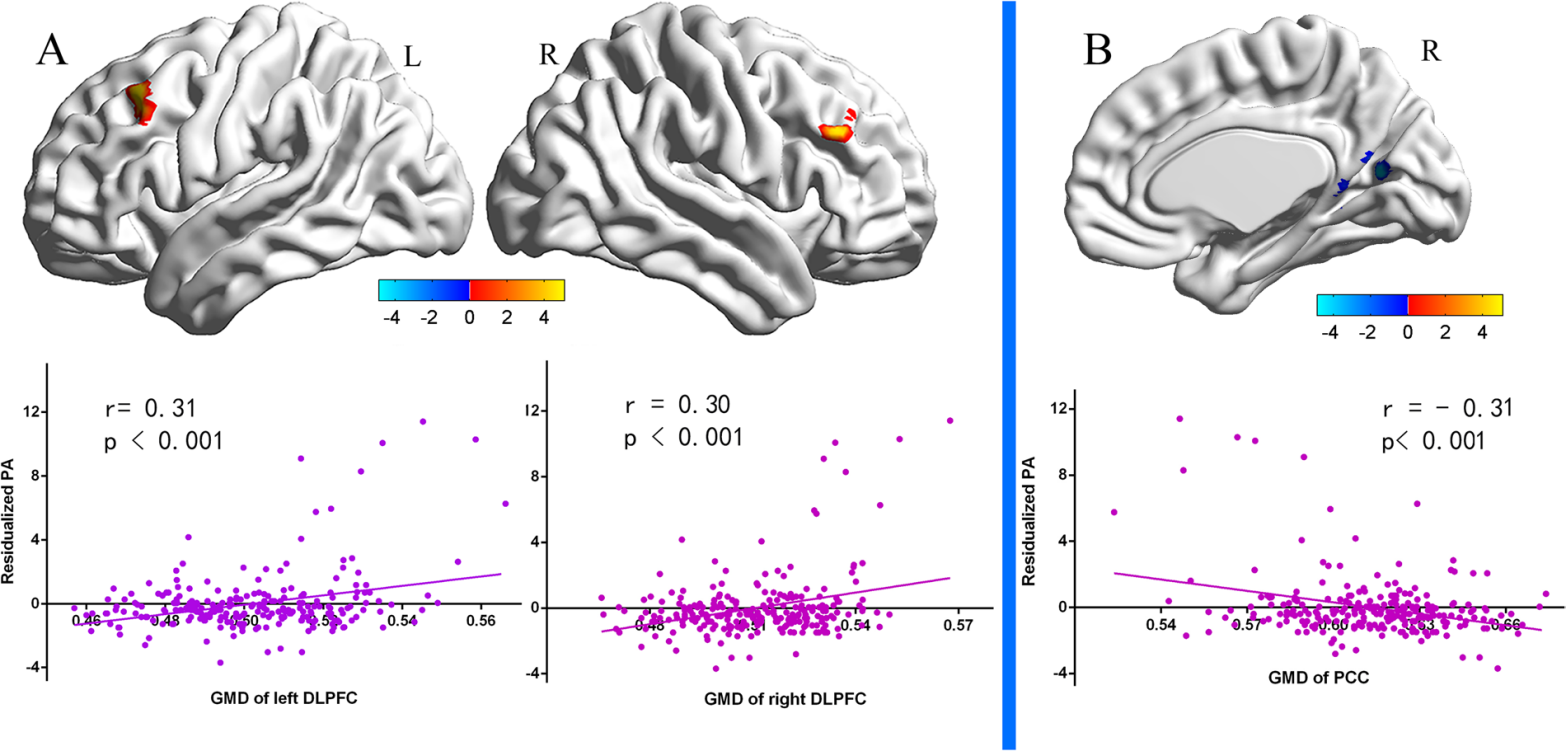
Correlations between regional grey matter density and proactive aggression. Scatter plots show the Pearson correlations between proactive aggression and GMD in the left DLPFC, right DLPFC and PCC, respectively, while reactive aggression scores were regressed out from proactive aggression scores. The scatterplots are shown for illustration purposes only. The threshold of the corrected cluster was set at p < 0.05 (single voxel p < 0.001, cluster size >219 voxels).

For the all participants, multiple regression analysis found that residual scores of proactive aggression were positively correlated with GMD in bilateral DLPFC (x, y, z = −41 24 45, *t* = 5.08; x, y, z = 48 32 32, *t* = 4.50), and were negatively correlated with GMD in posterior cingulate cortex (PCC, x, y, z = 6, −65, 14, t = −5.08, see Table 2, Fig. 1).

**Table 2:** Brain regions with significant correlations between rGMD and trait proactive aggression.

Brain regions	Peak coordinates	Cluster size	Peak T value
	x y z		
**Positive correlation**			
L-DLPFC	−41 24 45	235	5.08
R-DLPFC	48 32 32	241	4.50
**Negative correlation**			
PCC	6 −65 14	637	−4.58

GMD indicates Grey Matter Density; DLPFC, dorsolateral prefrontal cortex; PCC, posterior cingulate cortex. The Alphasim correction was conducted (The threshold of corrected cluster was set p < 0.05. Single voxel was set at p < 0.001. Cluster size >219 voxels).

The prediction analysis was then carried out to examine the stability of the relation between regional GMD and trait proactive aggression in all participants. The GMD in left DLPFC [r_(predicted, observed)_ = 0.18, p < 0.001, 1-*β* = 0.80], right DLPFC [r_(predicted, observed)_ = 0.26, p < 0.001, 1-*β* = 0.98] and PCC [r_(predicted, observed)_ = 0.31, p < 0.001, 1-*β* = 0.99] significantly predicted residual scores of trait proactive aggression.

Multiple regression analysis found that residual scores of reactive aggression were positively correlated with GMD in superior temporal gyrus (STG; x, y, z = 50, −44, 23, *t* = 4.33, p < 0.001, clusters > 50 voxels, uncorrected, see Table 3). We then carried out prediction analysis to confirm the relation between regional GMD in STG and residual scores of trait reactive aggression by machine learning method. The GMD in STG significantly predicted residual scores of trait reactive aggression [r_(predicted, observed)_ = 0.23, 1-*β* = 0.95, p < 0.001].

**Table 3:** Brain regions with significant correlations between rGMD and trait reactive aggression.

Brain regions	Peak coordinates	Cluster size	Peak T value
	x y z		
**Reactive aggression**			
**Positive correlation**			
STG	50 −44 23	80	4.33
**Negative correlation**			
______			

GMD indicates Grey Matter Density; STG, Superior Temporal Gyrus. The result was Uncorrected (Single voxel p < 0.001, Cluster size >50 voxels).

For the participants who did not score 0 for proactive aggression, multiple regression analysis found that residual scores of proactive aggression was positively correlated with GMD in bilateral DLPFC (x, y, z = −32 36 45, *t* = 4.03; x, y, z = 39 23 54, *t* = 5.77), and was negatively correlated with GMD in posterior cingulate cortex (x, y, z = 9–66 11, t = −4.83, see Supplementary Table S1 and Supplementary Fig. S1).

### Functional networks associated with trait proactive and reactive aggression

To explore whether the identified brain regions in the GMD analysis function synergistically with other brain regions to predict trait proactive aggression, a multiple regression analysis was performed. The significant brain regions (left DLPFC, x, y, z = −41 24 45; right DLPFC, x, y, z = 48 32 32; PCC, x, y, z = 6–65 14) in the GMD analysis were set as seeds in the functional connectivity.

**Figure 2:**
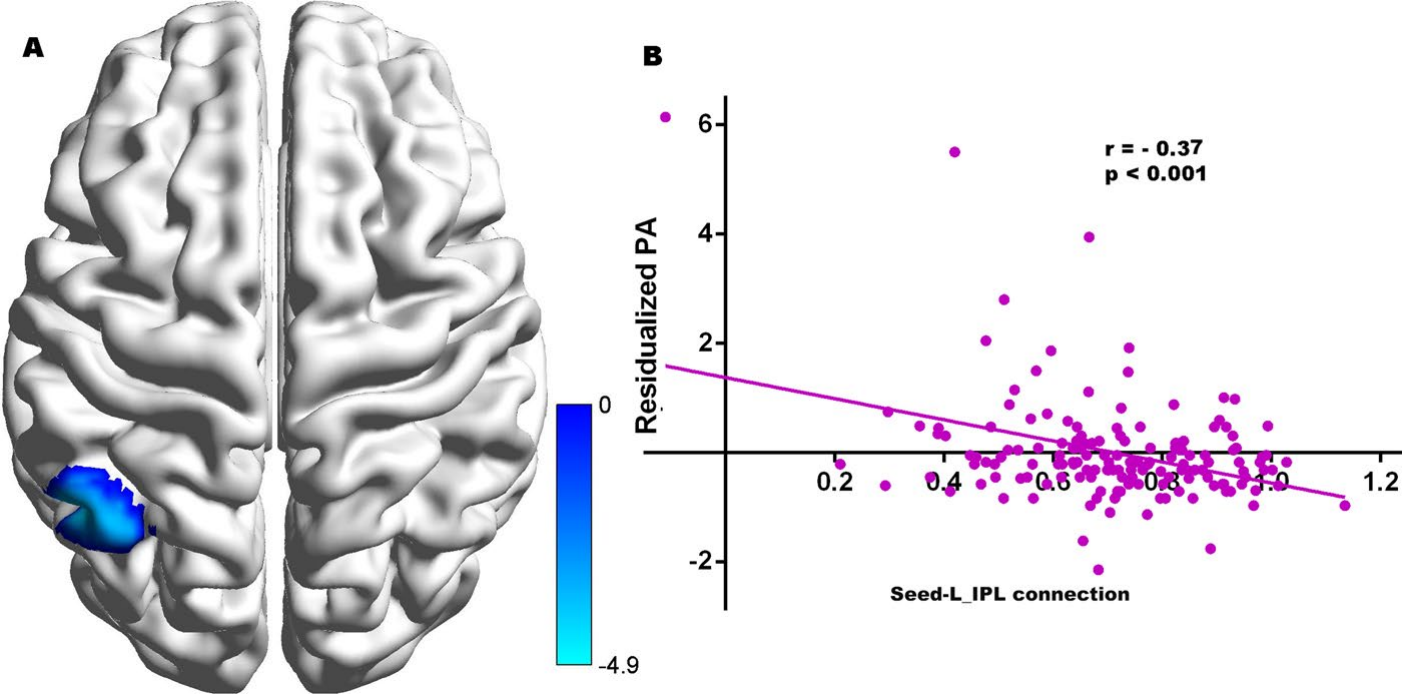
Clusters in which functional connectivity strengths with the left DLPFC (seed) were significantly associated with proactive aggression (Panel A). Colour bars represent t-values. Panel B indicates significant correlations between proactive aggression and functional connectivity strength between the left DLPFC and left IPL. The scatterplot is shown for illustration purposes only. The threshold of the corrected cluster was set at p < 0.05 (single voxel p < 0.001, cluster size >83 voxels).

**Figure 3:**
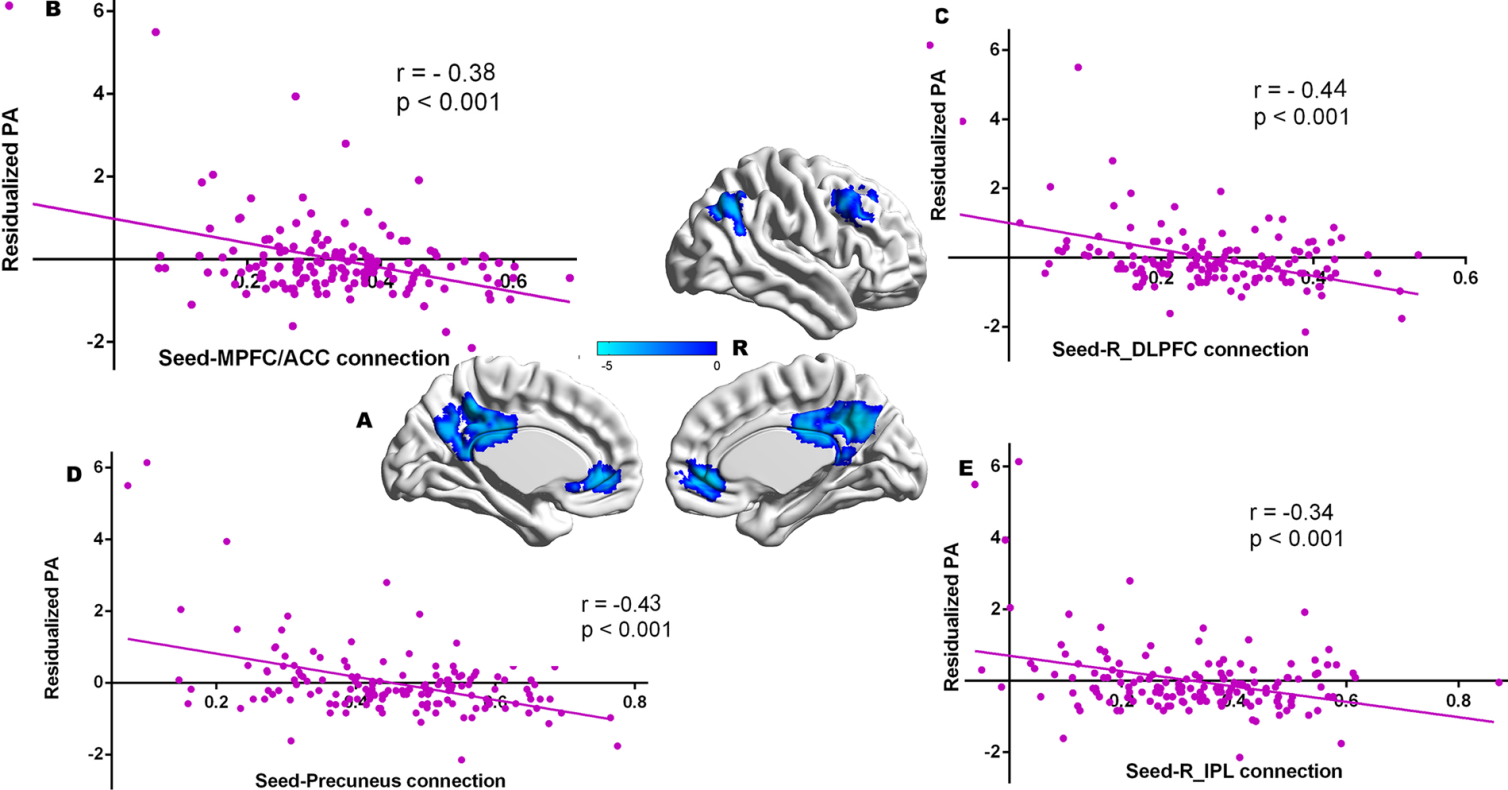
Clusters in which the strength of functional connectivity with the PCC (seed) were significantly correlated with proactive aggression (Panel A). Colour bars represent t-values. Scatter plots (panels B–E) indicate a significant association between proactive aggression and functional connectivity strength between the PCC and MPFC/ACC (panel B), right DLPFC (panel C), precuneus (panel D), and right IPL (panel E). The scatterplots are shown for illustration purposes only. The threshold of the corrected cluster was set at p < 0.05 (single voxel p < 0.001, cluster size >115 voxels).

For all participants who have resting data, with left DLPFC as the seed brain region, the residual scores of proactive aggressions were negatively correlated with strength of functional connectivity between left DLPFC and left IPL (x, y, z = −45–57 42, t = −4.89, see Table 4, Fig. 2). With the right DLPFC as the seed brain region, the residual scores of proactive aggressions were not significantly correlated with strength of functional connectivity between right DLPFC and any brain region. With PCC as the seed brain region, the residual scores of proactive aggression were negatively associated with the strength of the functional connectivity between the seed and the following regions: MPFC/ACC, precuneus, DLPFC (x, y, z = 6 45 −3, t = −4.93; x, y, z = 9–63 33, t = −5.03; x, y, z = 36 15 42, t = −5.15) and inferior parietal lobes (IPL, x, y, z = 48–57 39, t = −3.71) (see Table 4, Fig. 3).

**Table 4:** Brain regions in which functional connectivity strengths with seeds were significantly related to proactive aggression in all samples.

Brain regions	Peak coordinates	Cluster size	Peak T value
	x y z		
**L DLPFC as the seed**			
IPL	−45 − 57 42	131	−4.89
**R DLPFC as the seed**			
____			
**PCC as the seed**			
MPFC/ACC	6 45 −3	315	−4.93
precuneus	9 −63 33	895	−5.03
IPL	48 −57 39	140	−3.71
DLPFC	36 15 42	289	−5.15

Note: DLPFC indicates dorsolateral prefrontal cortex; MPFC, medial prefrontal cortex; ACC, anterior cingulate cortex; IPL, inferior parietal. The Alphasim correction was conducted (The threshold of corrected cluster was set p < 0.05. Single voxel was set at p < 0.001. Cluster size >83 and 115 voxels).

We then performed prediction analysis to examine the stability of the relation between RSFC and trait proactive aggression in the sample. The strength of the functional connectivity between left DLPFC and IPL significantly predicted residual scores of trait proactive aggression [r_(predicted, observed)_ = 0.28, 1-*β* = 0.94, p < 0.001]. The strength of the functional connectivity between PCC and the regions including MPFC/ACC [r_(predicted, observed)_ = 0.29, 1-*β* = 0.96, p < 0.001], precuneus [r_(predicted, observed)_ = 0.32, 1-*β* = 0.98,p < 0.001], DLPFC [r_(predicted, observed)_ = 0.37, 1-*β* = 0.99, p < 0.001], IPL [r_(predicted, observed)_ = 0.31, 1-*β* = 0.98, p < 0.001] significantly predicted residual scores of trait proactive aggression.

To explore whether the identified brain region (right STG, x, y, z = 50–44 23, t = 50–44 23) in the GMD analysis function synergistically with other brain regions to predict trait reactive aggression, a multiple regression analysis was performed. The significant brain regions in the GMD analysis were set as seeds in the functional connectivity. With STG as the seed brain region, after controlling age and gender, multiple regression analysis revealed that residual scores of reactive aggressions were not significantly correlated with functional connectivity strength between STG and any region.

For the participants who did not score 0 for proactive aggression, with left DLPFC as the seed brain region, the residual scores of proactive aggressions were negatively correlated with strength of functional connectivity between left DLPFC and left IPL (x, y, z = −48, −57, 42, t = −3.35, uncorrected, *p* < 0.001, 50 voxels). With the right DLPFC as the seed brain region, the residual scores of proactive aggressions were not significantly correlated with strength of functional connectivity between right DLPFC and any brain region. With PCC as the seed brain region, the residual scores of proactive aggression were negatively associated with the strength of the functional connectivity between the seed and the following regions: MPFC/ACC, precuneus, DLPFC (x, y, z = 9 45–3, t = −4.35; x, y, z = 15–60 33, t = −4.93; x, y, z = 36 12 42, t = −4.08, see Supplementary Table S2, Supplementary Fig. S2).

### Interaction effects between sex and proactive aggression on brain structural correlation and functional connectivity

After controlling for the effects of age and mean FD, the voxel-wise ANCOVA revealed no significant interaction effects between sex and residual scores of proactive aggression scores in terms of the GMD and the strength of RSFC with the identified brain regions in both all samples and the samples who did not score 0 for proactive aggression.

### Interaction effects between sex and reactive aggression on brain structural correlation and functional connectivity

After controlling for the effects of age and mean FD, the voxel-wise ANCOVA revealed no significant interaction effects between sex and residual scores of reactive aggression scores in terms of the GMD and the strength of RSFC with the identified brain region.

## Discussion

In this study, we investigated the brain correlates of individual differences in trait proactive and reactive aggression by combining structural (GMD) and functional (RSFC) approaches. Current study showed that residual scores of trait proactive aggression were positively related to the GMD in the bilateral DLPFC and negatively related to the one in the PCC. Additionally, we found that the functional connectivity between the left DLPFC and the IPL was negatively correlated with residual scores of proactive aggressions. Moreover, the strength of the functional connectivity between PCC and some brain regions, including bilateral DLPFC, bilateral IPL, ACC/MPFC, and precuneus, was negatively correlated with residual scores of trait proactive aggression. The results support that individual differences in trait proactive aggression relate to morphology and connectivity of some brain areas such as DLPFC and PCC. The details are provided in the following paragraphs.

First, as expected, the GMD of DLPFC was correlated with residual score of trait proactive aggression, suggesting that DLPFC may play an important role in proactive aggressive motivation (approach motivation towards instrumental goals via aggressive means) and the ability or tendency of behaviour monitoring (i.e., the ability of executive control of aggressive cognition and behaviour). As we illustrated in the introduction, individuals with high trait proactive aggression have high approval motivation and should be good at regulating cognitive conflict between benefit and morality, integrating information relevant to goal pursuit and using information guide behaviors in accordance with motivational goals^30^. Individual difference in grey matter volume in DLPFC is involved in one’s ability to exert control of dietary behaviours^45^. Additionally, DLPFC can flexibly encod specific attributes according to current goals^46^. Thus, DLPFC may represent the brain structure basis underlying individual differences in proactive aggressive motivation and the ability of cognitive regulation and control.

However, our results seem to be inconsistent with the previous study^24^, which found that trait proactive aggression scores were negatively correlated with adolescents’ volumes and cortical thickness of MFG, an area anatomically close to DLPFC. The inconsistence may be due to developmental changes in neural basis of behaviors^47^. For example, there are differential patterns of brain activation for the same task in subgroups at different ages^48^. Specifically, cognitive performance measured by a Stroop task was positively correlated with parietal activation during adolescence, whereas cognitive performance measured by the same task was positively correlated with prefrontal activation during adulthood^49^.

Second, as predicted, the GMD of the PCC was negatively associated with trait proactive aggression, suggesting that PCC may be related to proactive aggression-related moral cognition and emotion. As discussed earlier, some abilities and tendencies of moral cognition and emotion (such as low empathy and callousness) play an important role in trait proactive aggression. Prior studies^50,51^ suggest that PCC may be the neural basis underlying these abilities and tendencies of moral emotion and cognition. For example, structural evidence has shown that patient with empathic deficits (conduct disorder and schizophrenia) have smaller grey matter volume in PCC than healthy subjects do^52–54^. And psychopathy (which include low empathy and callousness) is negatively associated with grey matter volume in PCC^55,56^ too. Additionally, PCC activity was found to be positively correlated with the sensitivity of a moral issue and evaluating the appropriateness of solutions to personal moral dilemmas^50,51^. Compared with promoting goals (e.g., making good things happen), preventing goal achievement (e.g., keeping bad things from happening) activates PCC more strongly^57,58^. In summary, PCC may be the neural basis of individual differences in moral cognition and the emotional aspects of trait proactive aggression.

Third, RSFC analysis found that trait proactive aggression is negatively related to the strength of functional connectivity between DLPFC and both IPL and PCC. These results are consistent with our perspectives that people with high level of trait proactive aggression must be good at or like relieving or reducing moral inhibition and easily justifying their proactive aggression. Harmful behaviours are moral events^59^, and proactive aggression is a typical immoral behaviour. People’s moral systems inhibit harmful behaviours for personal interests^1,27^. Thus, the intentions or behaviours related to proactive aggression would be inhibited by moral systems, and the ability or tendency of moral disinhibition (e.g., ignoring negative moral outcomes and moral disengagement) can facilitate proactive aggression. As mentioned above, DLPFC plays a critical role in the ability or tendency of behaviour monitoring. The IPL plays a critical role in calculating the social cost of harming others^27^. More importantly, The connectivity between IPL and DLPFC may reflect individuals’ other-regarding tendencies^60^; IPL and PCC have been found to be involved in moral emotion (e.g., guilt and pain empathy)^61–63^. Presumably, the weaker strength of functional connectivity between DLPFC and IPL, PCC for high progressively aggressive people may reflect the brain network basis of the ability or tendency of moral disinhibition in trait progressive aggression, by which high proactive aggressive individuals more easily ignore victims’ anticipated pain or loss and negative emotions and outcomes resulting from aggressive behaviour.

**Figure 4:**
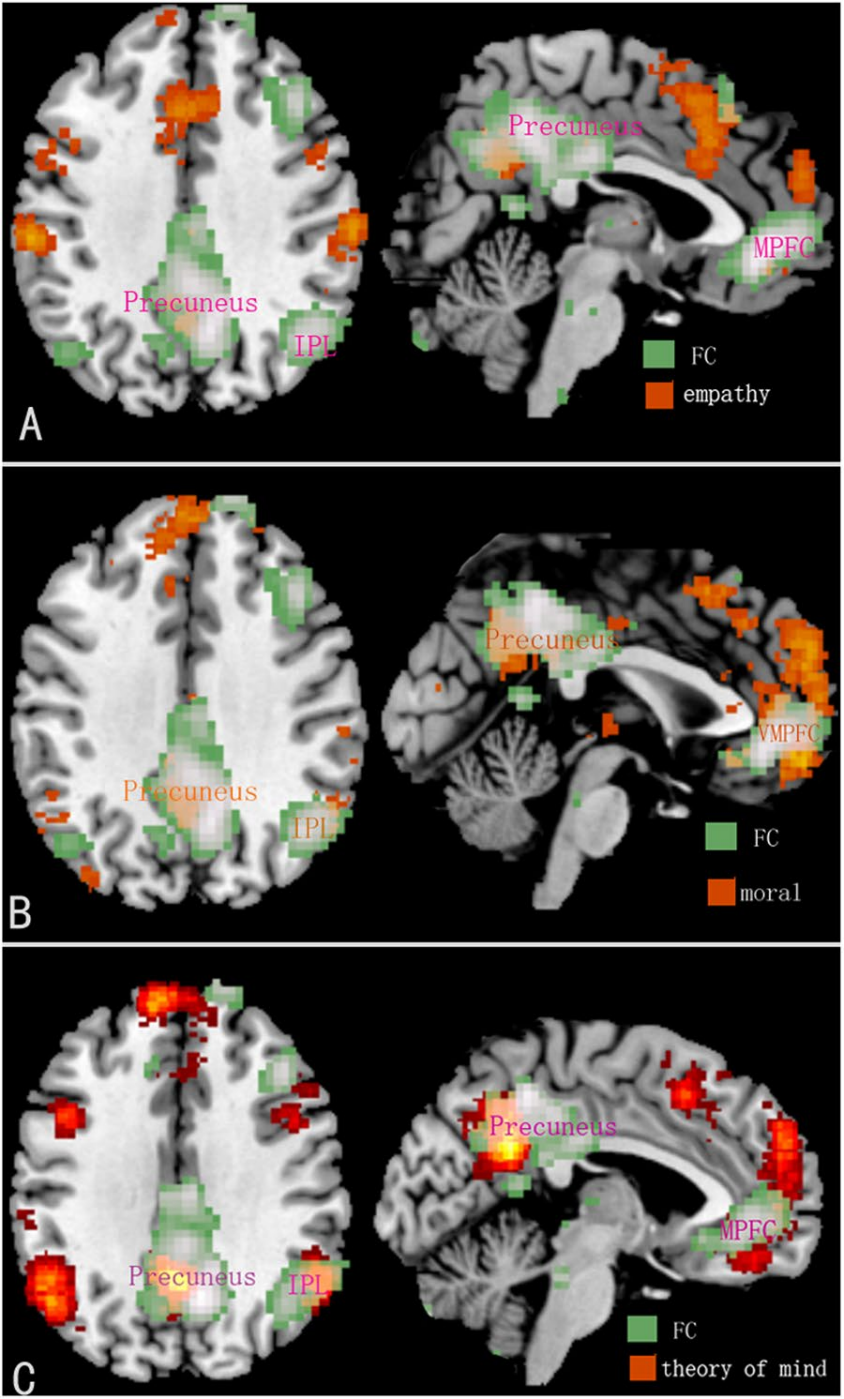
The overlaps between results of functional connectivity and simple meta-analysis from empathy (Panel A), morality (Panel B), and the theory of mind (Panel C) via Neurosynth.

In addition, RSFC analysis found that trait proactive aggression was negatively associated with the strengths of functional connectivity between PCC and bilateral IPL, MPFC/ACC, precuneus. These regions and coupling among PCC, bilateral IPL, MPFC/ACC, precuneus involved most of the regions and connections from the brain’s default model network (DMN)^64–66^. Presumably, not only the regions and coupling among PCC, bilateral IPL, MPFC/ACC, precuneus, but the DMN may be linked to trait proactive aggression. The DMN plays an important role in moral psychology, such as morality (e.g., guilt^67^), and the theory mind^44,68–72^ and empathy^73^. For example, participants with low empathy showed lower functional connectivity of MPFC/ACC within DMN, compared with a medium-empathy group^74^. Regions of DMN including PCC, ACC, and MPFC, are related to the moral self, which originated from moral identity and is defined as “the perception of a persons’ self as a moral person^75^. We examined overlap between results of functional connectivity and simple meta-analysis from morality, empathy and the theory of mind via Neurosynth (http://neurosynth.org). The overlaps supported the results that functional connectivity may be involved in moral cognition and empathy (see Fig. 4). Therefore, the DMN, especially PCC, MPFC/ACC, bilateral IPL, and precuneus, may be another neural basis underlying the moral cognition and empathy aspects of trait proactive aggression.

We observed that trait reactive aggression was positively correlated with the GMD in STG. Functional connectivity analysis did not find any regions that were associated with reactive aggression either. The finding regarding STG is consistent with previous studies that emotion processing is key component of reactive aggression^9,18^, and brain region related to emotion processing^76–78^ may be the neural basis of reactive aggression.

Although the results of current study support our hypotheses put forward in Introduction, there are some limits that must be acknowledged. First, we did not directly compare the neural basis of proactive aggression with reactive aggression because the results from the reactive aggression analysis was not significant. Future studies are needed to explore the neural basis of trait reactive aggression and replicate the current results. Second, the inference regarding the relationship between the neural substrates and the mental components of trait reactive and proactive aggression remains limited. Future studies should further examine the neural mechanisms of trait reactive and proactive aggression via longitudinal designs and using the specific measurements for these mental components. Third, this study is the first to discover the neural basis of trait proactive aggression. However, only young healthy college students with a high level of education were enrolled in this study. The results need to be replicated in other samples with different levels of education and with patients having specific diseases.

## Conclusion

In the present study, we combined structural (GMD) and functional (resting-state functional connectivity) analyses to examine the neural substrates of reactive and proactive aggression. The findings suggest that trait reactive and proactive aggression may have brain bases. Specifically, trait reactive aggression was positively correlated with GMD in STG. More importantly, DLPFC may serve as the brain structural basis of the trait proactive aggression components of proactive aggressive motivation and the ability of proactive aggressive cognitive regulation and behaviour monitoring. PCC, the functional connectivity between DLPFC and both IPL and PCC, and the functional connectivity between PCC and other brain regions, including MPFC/ACC, bilateral IPL, and precuneus, may be the brain bases of moral cognition and emotion components of trait proactive aggression. These findings suggest trait proactive aggression may be correlated with multiple components including approval motivation, moral cognition and emotion.

## Method

### Participants

In current study, 240 healthy, right-handed college students (112 males; mean age = 20.32, SD = 1.95) from Southwest University in China participated as part of our ongoing project to explore the association between aggression and mental health with brain imaging. None of them had a history of psychiatric or neurological disorders. All of the 240 participants were included in the VBM analysis. However, only 162 of them were scanned for the resting-state MRI. Seven of these participants were excluded due to excessive head motion (translational or rotational parameters >3 mm), resulting in 155 participants (62 males; mean age = 19.85, SD = 1.57) included in the RSFC analysis. All the participants completed the Reactive-Proactive Aggression Questionnaire (RPQ; Raine *et al*., 2006) after scanning. They provided informed consent and were paid for their participation. The experimental protocol was approved by the Southwest University Brain Imaging Center Institutional Review Board. The experimental protocol was performed in accordance with the standards of the Declaration of Helsinki.

### Reactive-proactive aggression questionnaire (RPQ)

The 23-item RPQ was used to measure to trait reactive-proactive aggression on a three-level scale^79^. In RPQ, 12 items assess responders’ proactive aggression (e.g., “Hurt others to win game”), and 11 items measure reactive aggression (e.g., “Angry when provoked”). Participants were asked to evaluate how often they carried out such behaviours or had these attitudes. The two-factor structure of the Chinese version of the RPQ was supported by confirmation factor analysis (RMSEA = 0.056, CFI = 0.96, TLI = 0.96), and it has good internal consistencies (reactive aggression, Cronbach’s α = 0.83; proactive aggression Cronbach’s α = 0.80) for assessing Chinese college students. In current study, internal consistencies were good for assessing both reactive aggression (Cronbach’s α = 0.83) and proactive aggression (Cronbach’s α = 0.83). Previous studies using RPQ revealed that scores of proactive aggression was correlated with the ones of reactive aggression^79–81^. In the current study, the correlation between scores of reactive aggression and the ones of proactive aggression was significant (*r* = 0.41, *p* < 0.001). To exclude the influence of reactive aggression on the neural basis of proactive aggression, residual scores of the proactive aggression scores regressing out reactive aggression^82^ were used in data analysis. Similarly, residual scores of reactive aggression regressing out proactive aggression were used to explore the neural correlates of individual differences in reactive aggression.

### Imaging data acquisition

All structural images were collected through a Siemens 3T Trio scanner (Siemens Medical, Erlangen, Germany). High-resolution T1-weighted anatomical images were acquired with a magnetization prepared rapid gradient echo sequence (repetition time = 1900 ms, echo time = 2.52 ms, inversion time = 900 ms, flip angle = 9°, thickness = 1 mm, slices = 176, resolution matrix = 256 × 256 mm^2^, and voxel size = 1 × 1 × 1 mm^3^). Structure images were collected within 4.5 minutes.

Resting-state functional images were collected using T2-weighted gradient-echo echo planar imaging (EPI) sequences (repetition time/echo time = 2000/30 ms, matrix = 64 × 64, flip angle = 90°, field of view = 220 mm × 220 mm, slice gap = 1.0 mm, slice thickness = 3 mm, acquisition voxel size = 3.4 × 3.4 × 4 mm^3^ and slices = 32). During the resting-state scanning, participants were instructed to relax and close their eyes but not fall asleep. The acquisition of functional images took approximately 8 minutes.

### Structural data pre-processing

Pre-processing steps of the brain structure analysis were conducted using SPM8 software (Wellcome Department of Cognitive Neurology, London, UK; www.fil.ion.ucl.ac.uk/spm/) that was implemented in MATLAB 2010a (Mathworks Inc., Natick, MA, USA). First, prior to the pre-processing, the original imaging data were converted into the format that were suitable for SPM8 and checked for artefacts or gross anatomical abnormalities to ensure the quality of the structural image. The origin of the coordinate of each image was manually reoriented to the anterior commissure. Second, images were segmented into white matter, grey matter and cerebrospinal fluid with New Segmentation in SPM8. Third, grey matter images for each participant were spatially normalized to a study-specific template using DARTEL (Diffeomorphic Anatomical Registration Through Exponential Lie algebra) tool^83^. In the later process, the average images of tissue segments were used to form an average template and each participant’s images were registered to the template. This process of registration was iterated such that individual participants’ new images resulted from normalizing each participant’s image to the previous averaging template were generated and a new template was then formed by averaging individual participants’ images. An optimal template was obtained, determined by minimising a measure of difference between the image and the warped template using a Levenberg–Marquardt strategy. Fourth, the optimal template was warped to the MNI space using the affine registration. Fifth, each participant’s grey matter and white matter images were normalized to the MNI space. Finally, the images were smoothed using 8 mm full-width at half maximum Gaussian kernel to enhance the signal-to-noise ratio.

### GMD-behaviour correlation analysis

Whole-brain analysis was conducted to explore the relationship between brain structure and trait proactive and reactive aggression. A multiple linear regression was performed between grey matter density (GMD) and residual scores of trait proactive/reactive aggression in the sample of all participants (n = 240), with gender, age, and total GMD as nuisance covariates. In this sample, the distribution of proactive aggression scores was skewed, and one hundred and forty students got zero scores for proactive aggression. To examine the influence of participants who scored 0 on brain correlates of proactive aggression, we conducted a multiple linear regression between residual scores of trait proactive and brain structure in the sample of participants (n = 100) who did not score 0 for proactive aggression using structural data, with gender, age, and total GMD as nuisance covariates. To effectively exclude noise, limit the search areas and avoid edge effects around the borders between grey matter and white matter, we used an absolute voxel signal intensity threshold masking of 0.2, ensuring that voxels with the probability of being grey matter lower than 0.2 would be excluded from the statistical analysis. A multiple comparison correction was performed using the AlphaSim program in REST software^84^. The threshold was set at cluster-level *P* < 0.05 and individual voxel level *P* < 0.001.

### Resting-state functional imaging data pre-processing

The data processing was conducted with SPM8 and Data Processing Assistant for Resting-State fMRI (DPARSF) software^84^. First, images from the first 10 time points were discarded to ensure fMRI signal stabilization. The remaining 232 volumes were corrected for slice order and head motion artefacts. Second, the images were spatially normalized to the MNI template with spatial normalization parameters. Subsequently, nuisance covariates, including the cerebrospinal fluid signal, white matter signal and Friston 24 motion parameters, were regressed out to eliminate the potential effect of physiological artefacts. Third, spatial smoothing with an isotropic 6 mm full-width at half-maximum (FWHM) Gaussian kernel was performed. Fourth, the linear trend was removed to reduce physiological noise (e.g., eye movements). Finally, a bandpass filter (0.01–0.1 Hz) was employed to reduce low-frequency drift and high-frequency noise^85^. Participants with the translational or rotational parameters that were greater than 3 mm (7 participants) and the mean framewise displacement (FD) values that exceed 0.3 (0 participant) were excluded from analysis. The mean FD values were derived using Jenkinson’s relative root mean square algorithm.

### RSFC-behaviour correlation analysis

To examine whether the clusters identified through the GMD-behaviour correlation analysis functioned with other regions as a network to explain trait proactive and reactive aggression, we performed RSFC-behaviour correlation analysis. First, the seed regions (left DLPFC, x, y, z = −41 24 45; right DLPFC, x, y, z = 48 32 32; PCC, x y z = 6, −65, 14; right STG, x, y, z = 50–44 23, t = 4.33) were defined using the coordinates of peak points of clusters identified in GMD-behaviour correlation analysis in the sample of 240 participants. Following previous studies^86,87^, we drew a radius sphere of 6 mm centred at these coordinates and extracted averaged time series for each seeds. We then examined the correlation coefficient between these seeds and the time series of all other voxels in the whole brain and transformed the correlation coefficient maps into z-maps using Fisher’s r-to-z transformation. Finally, at the group-level, we conducted a multiple linear regression analysis to identify the regions in which strength of functional connectivity to the seeds in z-maps was correlated with residual scores of trait proactive aggression in the sample of all participants that had resting data (n = 155), with age, gender and FD as nuisance covariates. AlphaSim was utilised for multiple comparison correction (corrected cluster-level *P* < 0.05 and individual voxel *P* < 0.001).

To examine the influence of participants who scored 0 on brain correlates of proactive aggression, we conducted a multiple linear regression analysis between residual scores of proactive aggression and strength of functional connectivity to the seeds in z-maps in both the sample of participants who did not score 0 for proactive aggression and had resting data (n = 65), with age, gender and FD as nuisance covariates. AlphaSim was utilised for multiple comparison correction (corrected cluster-level *P* < 0.05 and individual voxel *P* < 0.001).

### Interaction effects between sex and proactive/reactive aggression on brain structural correlation

In order to further examine sex effect on the brain basis of proactive/reactive aggression, we investigated whether the relationship between proactive aggression and structural correlation differed between the sexes in both the all participants and the participants who did not score 0 for proactive aggression, and whether the relationship between reactive aggression and structural correlation differed between the sexes in all samples. We conducted a voxel-wise analysis of covariance (ANCOVA) in SPM8, in which gender was defined as a group factor. Three covariates (age, gender total GMD) were included in the model and residual scores of proactive/reactive aggression scores were interacted with gender using the interactions option in SPM8. We assessed these interaction effects using t-contrasts.

### Interaction effects between sex and proactive/reactive aggression on functional connectivity

We investigated whether the relationship between residual scores of proactive aggression and RSFC with the selected seeds differed between the sexes in both the all participants who had resting data and the participants who did not score 0 for proactive aggression and had resting data, and the relationship between residual scores of reactive aggression and RSFC with the selected seed differed between the sexes in all participants who had resting data. Three covariates (age, gender, mean FD) were included in the model and residual scores of proactive/reactive aggression were interacted with gender using the interactions option in SPM8. We assessed these interaction effects using t-contrasts.

### Prediction analysis

To confirm the robustness of the brain-trait proactive aggression relationship, we implemented a machine learning approach, which is based on balanced cross-validation with linear regression^88–90^. Mean GMD and RSFC values were extracted for each cluster identified in GMD-behaviour and RSFC-behaviour correlation analysis using REX. In the regression model, the mean GMD or RSFC values of different regions obtained from the GMD and RSFC analyses were input as independent variables, and residual scores of proactive (reactive) aggression after regressing out reactive (proactive) aggression scores were dependent variables. The data was randomly and equally divided into four folds to ensure the distributions of independent variables and dependent variables across folds were balanced. Subsequently, three folds were employed to build a linear regression model and one-fold was left out. The model was used to predict the left-out fold data. This procedure was repeated four times, and the average correlation coefficients between the observed data and the predicted data (r_(predicted, observed)_) was obtained. The r_(predicted, observed)_ measures how well the dependent variables are predicted by the independent variable. Nonparametric testing was employed to examine the statistical significance of the model. One thousand surrogate datasets were generated to estimate the empirical distribution of r_(predicted, observed)_, against the null hypothesis that no correlation between trait proactive aggression or reactive aggression and regional GMD or RSFC. Each surrogate data set (D_i_) of size equal to the observed data set was generated via permuting the labels at the observed variables points (i.e. scores of proactive aggression). Then we calculated the r_(predicted, observed)_ of D_i_ (i.e., r_(predicted, observed)i_) with the actual D_i_ labels and the predicted labels using the four-fold balanced cross-validation procedure. This procedure produced a null distribution of r(predicted, observed)_i_ for the regression model. The statistical significance (p-value) of the correlation between the independent variables (GMD and RSFC value) and dependent variables (proactive/reactive aggression) was determined by the number of r (predicted, observed)_i_ values greater than r (predicted, observed) dividing the number of Di datasets (1,000)^89,91,92^. Finally, we used G*Power software (http://www.gpower.hhu.de) to calculate the statistical power of the prediction analysis in all samples.

## Supplementary information

Appendix

## Data Availability

The datasets generated during the current study are available from the corresponding author on reasonable request.
