# Supplementary material for: Brain structures and functional connectivity associated with individual differences in trait proactive aggression
Source: Sci Rep. 2019 May 22;9:7731. doi: 10.1038/s41598-019-44115-4 (PMC6531458; doi:10.1038/s41598-019-44115-4)
Supplement: Supplementary file 1 — Appendix [file 41598_2019_44115_MOESM1_ESM.docx]

**Brain structures and functional connectivity associated with**

**individual differences in trait proactive aggression**

Wenfeng Zhu^a^, Xiaolin Zhou^b,c,d *^, Ling-Xiang Xia^a*^

^a^ Research Center of Psychology and Social Development, Southwest University, Chongqing 400715, China

^b^ School of Psychological and Cognitive Sciences, Peking University, Beijing 100871, China

^c^ Beijing Key Laboratory of Behavior and Mental Health, Peking University, Beijing 100871, China

^d^ PKU-IDG/McGovern Institute for Brain Research, Peking University, Beijing 100871, China

Word Count of main text: 7069

Word Count of abstract: 249

Number of manuscript pages: 40

Number of tables: 3

Number of figures: 1

*Address correspondence to:*

Dr. Xiaolin Zhou

Department of Psychology

Peking University

Beijing 100871, China

Dr. Ling-Xiang Xia

Research Center of Psychology and Social Development,

Faculty of Psychology

Southwest University

Chongqing 400715, China

E-mail: xz104@pku.edu.cn; [xialx@swu.edu.cn](mailto:xialx@swu.edu.cn)

Tel: +86 23 6836 7841

Table S1 Brain regions with significant correlations between rGMD and trait proactive aggression in the samples who did not score 0 for proactive aggression.

| Brain regions | Peak coordinates | Cluster size | Peak *T* value |
| --- | --- | --- | --- |
|  | *x y z* |  |  |
| **Positive correlation** |  |  |  |
| L-DLPFC | -32 36 45 | 52 | 4.03 |
| R-DLPFC | 44 33 41 | 91 | 4.23 |
| R-DLPFC | 39 23 54 | 155 | 5.77 |
| **Negative correlation** |  |  |  |
| Ligual Gyrus | 24 -62 -8 | 78 | -4.80 |
| MOG | 41 -69 -2 | 66 | -4.20 |
| PCC | 9 -66 11 | 1182 | -4.83* |

Note: DLPFC indicates dorsolateral prefrontal cortex; MOG, middle occipital gyrus; PCC, posterior cingulate cortex. All T-scores reflect a threshold of p < 0.001 (uncorrected) and a minimum cluster size (k) of 50 contiguous voxels.

*Mean the Alphasim correction was conducted (The threshold of corrected cluster was set p<0.05. Single voxel was set at p < 0.001, cluster size >260).

Table S2 Brain regions in which functional connectivity strengths with seeds were significantly related to proactive aggression in the samples who did not score 0 for proactive aggression.

| Brain regions | Peak coordinates | Cluster size | Peak *T* value |
| --- | --- | --- | --- |
|  | *x y z* |  |  |
| **L DLPFC as the seed** |  |  |  |
| IPL | -45 -57 39 | 61 | -3.91 |
| **R DLPFC as the seed** |  |  |  |
| **____** |  |  |  |
| **PCC as the seed** |  |  |  |
| MPFC/ACC | 9 45 -3 | 119 | -4.35* |
| Precuneus | 15 -60 33 | 113 | -4.93* |
| Thalamus | 18 -30 3 | 65 | -4.06 |
| DLPFC | 36 12 42 | 98 | -4.08* |

Note: DLPFC indicates dorsolateral prefrontal cortex; MPFC, medial prefrontal cortex; ACC, anterior cingulate cortex. All T-scores reflect a threshold of p < 0.001 (uncorrected) and a minimum cluster size (k) of 50 contiguous voxels.

*Mean the Alphasim correction was conducted (The threshold of corrected cluster was set at p<0.05. Single voxel was set at p < 0.001, cluster size > 97).


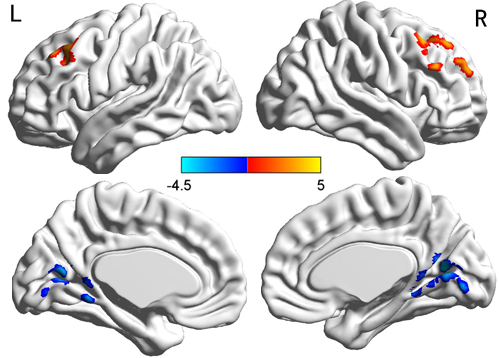


Fig. S1 Correlations between regional grey matter density and proactive aggression. The uncorrected clusters were presented at single voxel p < 0.005, cluster size > 400 voxels.


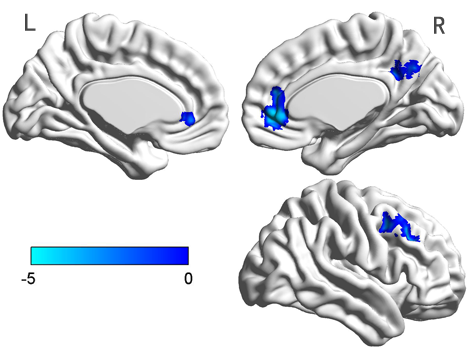


Fig. S2 Clusters in which the strength of functional connectivity with the PCC (seed) were significantly correlated with proactive aggression. The threshold of the corrected cluster was set at p<0.05 (single voxel p < 0.001, cluster size > 97 voxels).
